# The effects of reading and watching fiction on the development of social cognition: a systematic review

**DOI:** 10.1590/1980-5764-DN-2023-0066

**Published:** 2023-12-11

**Authors:** Júlia Ferreira Rezende, Nadia Shigaeff

**Affiliations:** 1 Universidade Federal de Juiz de Fora, Departamento de Psicologia, Juiz de Fora MG, Brazil.; 2 Universidade Federal de Juiz de Fora, Núcleo Interdisciplinar em Pesquisa em Neuropsicologia e Gerontologia, Juiz de Fora MG, Brazil.

**Keywords:** Social Cognition, Theory of Mind, Empathy, Motion Pictures, Reading, Cognição Social, Teoria da Mente, Empatia, Filmes Cinematográficos, Leitura

## Abstract

Social cognition is an umbrella term used to address the set of neurocognitive processes involved in effective social interaction, such as Theory of Mind and empathy, and is important for understanding of others’ intentions and actions and decision making. Narratives can serve as tools for learning social norms and understanding other people, as they involve mental simulations of social interactions. This review aimed to gather the results of current studies on the effects of reading and watching fiction movies on the development of social cognition. We included 16 publications, all of which were empirical studies. The results showed that, depending on individual factors, as well as on the specifics of the intervention, both reading and watching movies seem to influence the processes of development of social cognition, especially if associated with concomitant or subsequent activities, such as discussions. More research is needed to understand the specific details of this relationship.

## RESUMO

Cognição Social é um termo usado para se referir a uma coleção de processos neurocognitivos, como Teoria da Mente e Empatia, envolvidos em interações sociais, e é fundamental para a compreensão das ações e intenções de outros indivíduos e para a tomada de decisões. Narrativas podem servir como ferramentas para o aprendizado de normas sociais e para a compreensão de outras pessoas, pois envolvem a simulação mental de interações sociais. Esta revisão teve como objetivo reunir os resultados de estudos atuais sobre os efeitos da leitura e dos filmes de ficção no desenvolvimento da Cognição Social. Foram incluídas 16 publicações, sendo todas estudos empíricos. Os resultados demonstraram que, dependendo de fatores individuais e de aspectos específicos das intervenções, a leitura e os filmes parecem influenciar processos de desenvolvimento da Cognição Social, especialmente se forem associados a atividades como discussões. Mais pesquisas são necessárias para esclarecer os detalhes dessa relação.

## INTRODUCTION

Social cognition is an umbrella term used to address the set of neurocognitive processes involved in effective social interaction^1^. Social cognition involves many different processes, but mainly emotion processing (broadly defined as perceiving and using emotions), social perception (decoding and interpreting social cues in others), theory of mind/mental state attribution (the ability to represent the mental states of others) and attributional style/bias (the way by which individuals explain the causes and make sense of social events or interactions)^2^. Joint attention also seems to be more relevant from a developmental point of view. Social cognition can also be divided into three main domains: social perception, social understanding and social decision making^3^. For the purpose of this paper, we will focus on two specific processes involved in social cognition: empathy and Theory of Mind (also referred to as mentalizing)^4,5^.

Mentalizing describes the ability to infer the mental states of others, including their beliefs, thoughts, intentions and feelings^3,4^. Certain aspects of mentalizing are present in early childhood, but others, such as the ability to understand that someone may have a different belief from their own, are developed from the age of four^4^. Interpreting the behavior of others in terms of their mental states is an important part of decision making during social interactions^4-6^.

Empathy, as it differs from the Theory of Mind, involves not only the recognition of mental and emotional states (cognitive component), but also the ability to share emotional experiences (affective component)^7^. It is not an emotional state, but a set of processes that occur in response to the observation of another person’s emotional state, whether positive or negative^6,8^.

The components of social cognition are important because they allow us to understand the intentions and actions of others, modifying our own accordingly. Therefore, it is critical for decision making in the social context, as well as for understanding its consequences and maintaining successful interpersonal relationships^3,4^. Social cognition also allows us to learn about the world through the experience of others, without the need of first-hand experience, and appears to be related to affective considerations and prosocial behavior^3,9,10^. Specifically, the empathy-attitude model claims that adopting the perspective of an individual leads to increased empathetic feelings for this individual and, consequently, generalized valuing of the group that individual is inserted in, culminating in a more positive attitude towards the group^9^.

Stories (or narratives) can also provide the experiences needed for a better understanding of the world. It is argued that social cognition is used to understand stories, just as it is used to understand reality^11,12^. Therefore, narratives can serve as tools for learning social norms and understanding other people, as they involve mental simulations of social interactions^11,13^. This idea is supported by Bandura’s social learning theory: readers can learn vicariously from the actions and consequences of characters in the story^12,14^.

Narratives can be constructed in different ways, such as text, television, podcasts or comics. For the purpose of this study, we will consider stories presented in fiction texts (books and short fiction works) and movies. Previous studies have investigated the influence of literature on different aspects, such as empathy, theory of mind, self-reflection and emotional development^15-17^. On the other hand, few studies have investigated the effects of movies or other types of media on cognitive aspects.

Considering the importance of social contact for the development of certain cognitive aspects, such as social cognition, it is crucial to assess how these aspects can be influenced. In this context, the evaluation of the effects and potential benefits of reading fiction and watching movies becomes especially significant. This assessment aims to determine whether these activities could facilitate the development of social cognition and potentially be as vital as direct social interaction. These investigations may also be important in developing alternative treatments and interventions for patients diagnosed with autistic spectrum disorder (ASD) and those diagnosed with schizophrenia. Both diagnoses are characterized by deficits in social functioning as one of the main diagnostic criteria, especially deficits in the ability to mentalize^18,19^. Studies by Gürcan et al.^18^, Peña et al.^20^ and Pino and Mazza^21^ suggest that interventions using literary fiction or movies can improve clinical symptomatology in schizophrenia, as well as promote mentalizing and social functioning skills. Similarly, studies performed with people with ASD suggest that reading could improve social understanding^22^ and film analysis interventions could enhance the development of perspective-taking abilities^23^.

A systematic review, also known as “research synthesis”, provides an overview of relevant studies to summarize current knowledge of the literature on a specific topic. Therefore, the general aim of this systematic review is to summarize the results of current studies on the effects of fiction reading and watching movies on the processes involved in the development of social cognition, such as empathy and the Theory of Mind. More specifically, we hope to:

- provide an overview of the most recent findings in the scientific literature regarding the impact of reading fictional works on the development of social cognition;
- present the outcomes of the latest scientific publications concerning the effects of watching movies on the development of social cognition;
- compare the results of studies involving the effects of reading fictional works on social cognition with those of studies involving the effects of watching movies on social cognition; and
- investigate the duration and intensity of observed effects, if any.

## METHODS

A search was performed from October 18^th^ to October 28^th^ of 2021. The Preferred Reporting Items for Systematic Reviews and Meta-Analyses (PRISMA) Statement guidelines were used as the methodology for this systematic review. The descriptors and their variations were chosen from the Health Sciences Descriptors (in Portuguese, *Descritores em Ciências da Saúde* — DeCS) research bases, for studies in Portuguese, and Medical Subject Headings (MeSH) for studies in English. The descriptors were: social cognition, reading, theory of mind, movies, and empathy. They were then combined to perform searches in the following databases: PubMed, Cochrane Library and Scientific Electronic Library Online (SciELO).

To be included in the study, publications must meet the following inclusion criteria:

- language: publications in English and Portuguese;
- time range: publications from 2011 to 2021;
- study design: empirical studies, such as evidence-based, qualitative analysis, observational or mixed methods;
- intervention: studies involving reading and movie interventions of all types: reading training, reading sessions, movie sessions, movie clip presentation.

The exclusion criteria were: book chapters, systematic reviews, case studies, monographs, dissertations or thesis. Interventions that involved non-fiction narratives or documentaries were also excluded.

The first search was performed in all data bases, resulting in 1,906 papers in PubMed, 712 in Cochrane Library and 15 in SciELO. Database filters were used to select only publications from 2011 to 2021. On the search on PubMed, a filter for language of publication was also used. The manuscripts were then evaluated by two independent reviewers based on the inclusion and exclusion criteria. If there was disagreement between the two reviewers, a third one was asked to assess the inclusion of the study. After removing duplicate studies, titles and abstracts were evaluated to determine whether the studies were pertinent to this literature review. In total, 34 publications were selected at this stage. Then, the manuscripts were read in full, in order to understand the findings and results of the interventions. Finally, studies were analyzed according to the following characteristics:

- setting and design,
- participants,
- intervention, and
- themes.

**Figure 1.**
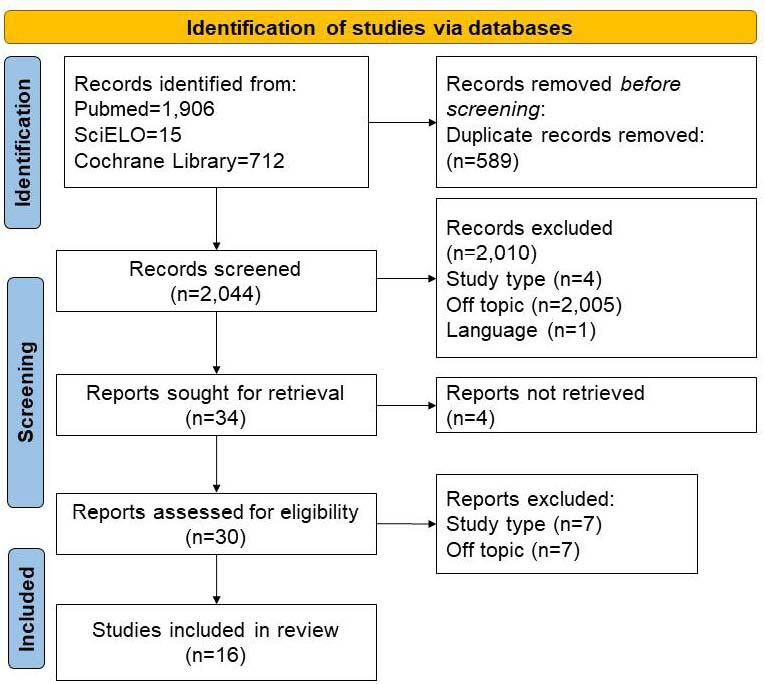
Flowchart of the studies review and selection process.

This process is shown in the flowchart in Figure 1.

## RESULTS

The initial search of all databases resulted in 2,633 studies, including 1,906 from PubMed, 15 from SciELO and 712 from Cochrane Library. After duplicity analysis, 589 publications were excluded. Therefore, 2,044 studies were selected for title and abstract analysis, from which 2,010 were excluded: 2,005 were outside the scope of this review due to the topics researched; four were excluded due to the study design; and one did not meet the inclusion criteria for the language. Twenty-eight studies were selected from PubMed, three were selected from SciELO and three from Cochrane Library to be read in full. After full reading, 14 studies were excluded and a total of 16 studies were approved for inclusion in this systematic review: 12 from PubMed, two from SciELO and two from Cochrane Library. The studies selected to be included in this review were all empirical studies that portray the assessment of interventions through fiction movies or fiction reading in order to improve social cognition, socio-cognitive abilities, theory of mind or empathy. Detailsabout the selected studies are summarized in Table 1.

**Table 1.** Description of the main results of the publications used in the systematic review.

Authors	Study type	Instruments	Participants	Intervention	Duration of intervention	Outcomes
Ahmadzadeh et al.^24^	Intervention	Jefferson Scale of Empathy-Health Professional Students	133 participants were distributed into four groups: A (n=42), B (n=23), C (n=22) and D (n=46)	A three-hour workshop (Group A); watching the movie “The Doctor” (Group B); watching the movie “The Doctor”, then, participating in a three-hour workshop the next day (Group C); control group with no intervention (Group D).	1 or 2 days, depending on the experimental group, and reassessment of empathy a month later	All of the three interventions had an immediate improving effect on empathy scores compared to the control group. One month later, only improvements on groups A and C remained significant.
Bal & Veltkamp^25^	Intervention	Emotional Transportation scale by Busselle and Bilandzic^39^ Empathic concern scale by Davis (1980;1983) Narrative Understanding Scale Attentional Focus Scale	66 students in Study 1 (36 in the fiction condition and 30 in the control condition) 97 students in Study 2 (50 in the fiction condition and 47 in the control condition)	Participants in the experimental condition read a fictional narrative and participants in the control group read a nonfiction piece	Participants read the book and responded the measures both immediately after and a week after	Emotional transportation was positively related to the empathy scores measured after a week, but not right after the intervention
Collins et al.^28^	Intervention	Jefferson Scale of Empathy – Health Profession Students (JSE-HPS)	21 participants were distributed into an intervention (n=11) or control group (n=10)	Participants of the experimental group read short pieces of literary fiction over the course of 8 weeks. No action was required of the students in the control group.	Participants read short excerpts weekly over the course of eight weeks.	Results showed a statistically insignificant increase in the scores of participants in the intervention group.
Dias-Corrêa et al.^34^	Intervention	Avaliação Sociocognitiva de Respostas Infantis Pós Exploração Dialogada de Narrativa Textual^*^ Questionário de Respostas Socialmente Habilidosas Segundo Relato do Professor (QRSH-RP)^†^ Brazilian version of the Strengths and Difficulties Questionnaire (SDQ)	45 children distributed into two groups, which received the same intervention in two different years (2010 and 2011), group 1 (n=25) and group 2 (n=20)	Reading of storybooks with a focus on mental states and emotions	Two or three sessions of 50 minutes weekly, from August to November, with a total of 25 sessions	Gains in children’s social cognitive abilities and development of emotion, thought, intention and behavior comprehension of the characters in the stories were observed.
Mani et al.^36^	Intervention	-	11 third-year undergraduate dental students	After analysing literary texts, students participated in perspective-taking activities, and reflected on the effects of the study.	Three months	After content analysis, three main themes/learning points: facilitate empathy, stimulate self-awareness and motivate perceptive communication.
Gürcan et al.^18^	Intervention	The Positive and Negative Symptoms Scale (PANSS), Reading the Mind in the Eyes Test (RMET) Montreal Cognitive Assessment Scale (MoCA) Dokuz Eylül Theory of Mind Index (DEToMI) Social Functioning Assessment Scale (SFAS)	28 schizophrenia patients, medicated and clinically stable, distributed into two intervention groups: narrative therapy (n =14) and movie therapy (n = 14)	Narrative therapy or movie therapy	Once a week for 14 weeks	A significant difference was found between PANSS negative, DEToMI, SFAS and RMET pre- and post-intervention mean scores in both groups, but RMET and DEToMI scores were higher in the MT group.
Hojat et al.^26^	Intervention	Jefferson Scale of Empathy-Health (JSE)	248 medical students divided into experimental group (n=129) and control group (n=119)	Students in the experimental group were shown the movie clips and then asked to discuss positive and negative effects of it. Ten weeks later, the students in the experimental group were divided: half of them participated in a lecture about empathy, while the other half watched a different movie.	1 hour of presentation plus discussion, with a gap of ten weeks between phase 1 and phase 2	A significant increase in empathy was observed in the experimental group after phase one, but the improvement in empathy score was sustained after phase 2 only in the experimental group, which participated in the empathy lecture.
Kidd & Castano^31^	Intervention	False-belief tests as a measure of cognitive ToM Reading the Mind in the Eyes Test (RMET) Diagnostic Analysis of Nonverbal Accuracy 2 - Adult Faces test (DANVA2-AF) Yoni test, which measures both cognitive and affective ToM Author Recognition Test	86 participants in experiment 1 (randomly assigned to read literary fiction or nonfiction) 114 participants in experiment 2 (randomly assigned to read literary fiction or popular fiction) 69 participants in experiment 3 (randomly assigned to read literary fiction or popular fiction, using different texts from experiment 2) 72 participants in experiment 4 (randomly assigned to read literary fiction or popular fiction, using the same texts from experiment 3 with two additional texts for each condition)	Participants read short passages of either nonfiction, literary fiction or popular fiction.	Participants read the passages and were evaluated immediately after.	Literary fiction did increase ToM and results on the Author Recognition Test predicted RMET scores.
Klemenc-Ketis & Kersnik^37^	Intervention	-	11 fourth-year medical students	Students participated in an elective course about professionalism in medicine for 4 months, which included the screening of movies.	Four months	Students recognised communication, empathy, doctors’ personal interests and palliative care as dimensions of the movie, and reported that these dimensions were important aspects of the medical practice.
Panero et al.^30^	Intervention	Reading the Mind in the Eyes Test (RMET) Author Recognition Test (ART)	792 participants (distributed into four conditions: reading of literary fiction, nonfiction, popular fiction or no reading) Replication of Kidd and Castano’s^31^ experiment	Participants read short passages of either nonfiction, literary fiction or popular fiction.	Participants read the passages and were evaluated immediately after.	Literary fiction had no significant effects on theory of mind, but results on the Author Recognition Test predicted RMET scores.
Peng et al.^33^	Intervention	Jefferson Scale of Empathy-Health Professional Students Semi-structured interview	45 second-year undergraduate nursing students	Participants watched a movie (“Still Alice”) and participated in a Virtual Dementia Tour (VTD).	The different steps of the intervention were performed in the course of three days	Participants’ empathy levels demonstrated significant overall improvements.
Pino & Mazza^21^	Intervention	Basic Empathy Scale - Cognitive Empathy Sub-scale (BES- CES) The Eyes Task (Revised “Reading the Mind in the Eye”) Interpersonal Reactivity Index cognitive sub-scales (IRI) Advanced Theory of Mind Task Attribution of Intentions Task Empathy Quotient Scale First and Second Order False Belief Test Multifaceted Empathy Test (MET) -Cognitive Empathy (CE) Faces Test Emotion Attribution Task Basic Empathy Scale-Affective Empathy Sub-scale (AES)	214 participants were distributed in the three experimental conditions (reading of literary fiction, nonfiction or science fiction).	Participants read literary fiction, nonfiction or science fiction books.	The time between the pre- and post-reading phases was 14 days, and the participants had one week to read the book.	Literary fiction facilitated changes indicative of improvement in mentalizing abilities.
Rodrigues et al.^32^	Intervention	Avaliação sociocognitiva de respostas infantis pós-exploração dialogada de narrativa textual^*^	57 first-grade children of a public school in Minas Gerais	Reading of storybooks with a focus on mental states and emotions.	Teacher training lasted 20 hours, distributed into three months.	Increase in childrens’ attribution of mental states and significant improvement in social cognitive abilities scores after the intervention.
Samur et al.^29^	Intervention	Reading the Mind in the Eyes Test (RMET) Author Recognition Test (ART) Bermond–Vorst Alexithymia Questionnaire Transportation Scale Osnabrück Life Stress Scale Adult Survey of Reading Attitudes	80 participants in experiment 1 (comparison between the effects of reading literary fiction and nonfiction on mentalizing) 80 participants in experiment 2 (comparison between the effects of reading literary fiction, nonfiction and popular fiction) 90 participants in experiment 3 (comparison between the effects of reading literary fiction, nonfiction, popular fiction and a control condition with no reading) 80 participants in experiment 4 (comparison between the effects of reading literary fiction, nonfiction, popular fiction and a control condition with no reading, using different texts from experiment 3)	Participants read short passages of either nonfiction, literary fiction or popular fiction.	Participants read the passages and were evaluated immediately after.	Higher RMET scores were observed on the literary fiction condition, but these findings were not statistically significant. Results on the Author Recognition Test predicted RMET scores.
Swami et al.^35^	Intervention	Scale of Ethnocultural Empathy	87 Malaysian citizens enrolled on undergraduate and postgraduate programs in three universities	Round Table Cinema Activity (RTCA)	Three different encounters, with two-week gaps in between	Participants’ ethnocultural empathy showed an increase following the intervention, and qualitative data analysis revealed a trend toward improvement in participants, driven by significant changes observed throughout the study.
Tompkins^27^	Intervention	Social Skills Improvement System (SSIS) Rating Scales False belief tasks Peabody Picture Vocabulary Test - 4th edition Adaptation of the Head Start Family and Child Experiences Survey	73 low-income children distributed into experimental, storybook control group and non-treatment group (distribution of children was not specified).	Children in the experimental group participated in one-on-one book reading interactions, including questions about mental states, introduction and embedded statements and summary of false beliefs in the story. The storybook control group was read the same storybooks without statements, questions or summary. Non-treatment control group engaged in no interaction with the experimenter during the training phase.	Each child received 8 to 15 storybooks on separate days, approximately 3 books per week over 5 weeks.	Significant improvements were observed in the children’s false belief understanding, as well as statistically insignificant changes in the social competence of the experimental group.

Notes: ^*^in a free translation to English, “Sociocognitive assessment of children’s responses after dialogued exploration of textual narrative”; ^†^in a free translation to English, “Questionnaire of Socially Skilled Responses, as reported by teachers”.

Of the 16 selected publications, four^24-27^ were randomized controlled trials in which participants were randomly distributed into groups, including a control group, and underwent pre- and post-intervention testing with additional follow-up testing some time later. One^28^ of the trials did not include a follow-up. Two other studies randomly allocated participants and included pre- and post-intervention testing^18,21^, but did not include a control or follow-up group, while three studies only presented post-intervention testing^29-31^. In addition, four studies were not randomized, but included pre- and post-tests^32-35^. Finally, two studies performed the qualitative analysis of the results^35,36^.

In most studies, intervention participants were predominantly female. Most of them were between the age of 20 and 30 years old, but some studies included a wider age range, including participants up to 70 years old. Most studies were performed with university students or randomly selected participants. Specific studies have focused on patients with schizophrenia and children.

The overall results revealed a predominance of research focused on the effects of reading fiction compared to watching fiction movies. The search also revealed that most of the studies that explored the relationship between social cognition and fiction stories were carried out in the past five years, especially with regard to the specific relationship between fiction movies and theory of mind and empathy. The geographic location of the selected studies were also very diversified, including Turkey, China, Iran, USA, Malaysia, Netherlands, Italy, Brazil and Slovenia.

Five of these studies^24,26,33,35,37^ focused on the possible effects of movie interventions on students of health professions, such as nursing and medicine. Only one of the described interventions^18^ was performed in patients with schizophrenia. The methodology in all of these interventions varied, but generally involved the presentation of a fiction movie followed by discussion, lecture, workshop or writing activities. It should be noted that only two^24,26^ out of four quantitative studies^18,24,26,33^ included a control group, as well as pre- and post-intervention testing and follow-up. The remaining two obtained their results predominantly through qualitative analysis^35,37^.

Overall, the results of the studies mentioned above showed that a combination of movie screening with a more direct learning activity, such as post-discussions or technical content, could be more effective at improving empathy than just a movie presentation. These results were more clearly observed in the studies performed by Ahmadzadeh et al.^24^ and Hojat el al.^26^, which investigated the combination of movie screening with follow-up activities, in comparison to a movie presentation alone. Likewise, this combination could provide more lasting effects on empathy. There also seemed to be no difference between a full movie and clip presentation. Instrument measures differed between studies, considering specific aims and locations, but the Jefferson Scale of Empathy (JSE) was used frequently.

With regard to publications about interventions with texts, passages or fiction books, the characteristics of the samples were diverse. Some of the studies selected their participants randomly^21,28-31^, some were performed with university students^25,36^ and three were focused on children between three and seven years old^27,32,34^.

One of the most relevant studies on the effects of reading fiction on social cognition was performed by Kidd and Castano^31^, who compared the effects of reading literary fiction with reading nonfiction, as well as with popular fiction. Their hypothesis was that literary fiction, due to its specific features, would engage Theory of Mind processes and promote it, but not popular fiction. Three of the selected studies^21,29,30^ attempted to replicate the findings of Kidd and Castano’s study. Different from the original study, two of the replication studies^29,30^ failed to find significantly higher theory of mind scores after reading literary fiction compared to any of the other conditions. Another study by Collins et al.^28^ obtained the same results. On the other hand, Pino and Mazza^21^ managed to find a significant increase in the scores of mentalizing in the literary fiction experimental group. It is noteworthy that in this study, unlike the previous ones, the participants read a short book in full, and not just excerpts from literary fiction, and a greater variety of instruments was used. It should also be noted that only one of these studies included a control group^28^, and only two^21,28^ performed pre- and post-intervention testing.

Instrument measures differed between studies, considering the specific aims of each of these studies, but the Reading the Mind in the Eye Test (RMET) and the Author Recognition Test (ART) were used quite frequently, along with specific empathy scales. A consistent finding in three^29-31^ of the above mentioned studies was that ART scores predicted RMET scores, suggesting that longer exposure to fiction may be more related to the theory of mind development than a one-time exposure to literary passages. However, a limitation of this finding is that ART does not differ between literary and popular fiction. Moreover, these studies did not include theory of mind measures prior to the intervention, and did not control for baseline individual differences.

Three studies explored the effects of reading on the development of social cognitive abilities in children^27,32,34^. In all of them, children participated in interventions in which storybooks focusing on mental states and emotions were read by a teacher or member of the research team. Interventions included discussions on the relevant aspects of the story. Improvement in social cognitive abilities, including empathy, as well as mental states comprehension, was observed across all studies, as measured by a variety of quantitative and qualitative instruments. Children in all studies were younger than seven and older than three years old, and only one of them^27^ assessed whether children had adequate and similar language skills before the intervention. It should also be noted that the social cognitive measure used in the study by Dias-Côrrea et al.^34^ was not validated, and the children were evaluated by teachers who knew the purpose of the study, which may suggest a risk of assessment bias. Likewise, the observation of teachers in the study by Rodrigues et al.^32^ was not systematic. In addition, only the study by Tompkins^27^ included a control group and follow-up measures.

Two studies^25,36^ differed in general patterns of interventions and varied in focus. Only one^36^ evaluated the effects of stories in various formats (including short stories, poems, memoirs and films) portraying the experience of pain and suffering, and obtained positive effects on participants — dental students —, such as facilitating empathy, stimulating self-awareness and motivating perceptive communication. Bal and Veltkamp’s^25^ randomized controlled trial, on the other hand, focused on the role of emotional transportation, considering that reading fiction would affect empathy over time only when the reader is emotionally transported into a story. This hypothesis has been confirmed, and is in line with insights from previous studies^21,29,30^, in which the authors suggest that the effect of reading on the theory of mind and empathy is dependent on individual characteristics of the reader.

The results obtained in this search are summarized in Table 1.

## DISCUSSION

The general results suggest that interventions performed with fiction books and movies can actually influence some of the social cognitive processes evaluated. On the other hand, both movies and literature seem to have more effect if associated with different subsequent or concomitant activities, such as discussions or lectures, both in the intensity and duration of the results. Similarly, reading per se, specifically reading literary fiction, appears to be mediated by a number of individual factors, such as emotional transportation, which refers to the feeling of being completely immersed in a story and temporary loss of awareness of self and the real world^38,39^. Content and features of the literary text (for example, literary genre), as well as aspects regarding the reader (for example, reflection process) also seem to have an influence^40^. Depending on these factors, the observed effects seem to vary.

Some studies also suggest that literary fiction is the only type of text capable of influencing theory of mind and empathy, but more studies should be conducted in order to fully understand the specific features of a so-called “literary” text, since this is still an uncertain concept and the distinction of literary genders seems to be dynamic^41^. While some studies considered the focus on characters as a feature of literary texts^21^, others considered that literary texts were prize-winning texts^31^. However, there is evidence that suggests the relationship between social cognition, specifically its aspect of morality, and different literary categories could be dynamic^42^.

Additionally, the correlation between ART scores and theory of mind measures was observed in all studies in which these variables were included. The ART assesses exposure to fiction, and these findings may indicate that the habit of reading fiction could exert a more substantial influence on social cognition processes^16^ than the immediate temporary effect of reading individual passages or short texts. However, on the other hand, it is also possible that individuals with better theory of mind performance are more inclined towards engaging in fiction reading. Future studies should also explore long-term effects of the habit of reading fiction, possibly through longitudinal studies.

This correlation may also suggest a reciprocal relationship between reading or watching fiction and social cognition^30^. Rather than peoples’ social cognitive abilities being improved by reading or watching fiction, it may actually be that people who are more interested in psychological states or who already have higher scores on Theory of Mind and empathy look for fictional texts or movies through which they can explore these aspects. In this case, the observed relationship would be a consequence of this inherent individual aspect and not of the exposure to fiction books or movies itself. Likewise, environmental aspects, such as insertion in university and chosen course, could influence the nature of this observed relationship, as well as the attitude of the participants towards the text^30,43^.

On the other hand, studies performed with children suggest an overall effect of reading interventions. I terventions with storybooks and mediated discussions of mental states demonstrated an overall increase in children’s sociocognitive abilities. This shows that even indirect forms of consumption of fictional stories could influence cognitive processes, if accompanied by guided discussions. These findings are especially important as they show how storybooks can be a tool for developing sociocognitive abilities in young children, which are critical for social interaction and behavior^4-6^.

In addition, many of the selected studies were conducted with medical/nursing students, which suggests that reading and movie interventions could be especially useful to develop empathy in these groups, given that social skills are very important for these professional practices. Likewise, these findings may also be useful for the development of innovative interventions aimed at patients with schizophrenia and ASD, considering the deficits in social cognition observed in these groups^18,44^.

It is noteworthy, however, that only four of the studies analyzed presented a rigorous methodology, aiming to reduce bias and more effectively examine the effects of the intervention. Although both literature studies^25,27^ and both movies studies^24,26^ reported promising results regarding positive intervention outcomes, future studies should consider this aspect to better understand the relationship between fiction stories interventions and the development of social cognition.

Overall, this systematic review focused on interventions aimed at improving social cognition performance. Considering that social cognition is an umbrella term, constructed by different components, in this review, the selected components were theory of mind and empathy. Although these components are indeed related and form part of a larger concept, a limitation of these studies, as well as of this review, is the wide range of terms and definitions, which can make it difficult to synthesize these findings. For example, only some of the studies recognized the difference between affective and cognitive empathy, while others did not. Other limitations include small samples, prevalence of female participants and lack of a control group in most studies. A limitation of this review is the lack of publications in other languages, such as Spanish, as well the limited number of manuscripts included, which may make it difficult to generalize the results.

Future studies should consider these variables, as well as other forms of narrative fiction construction, such as TV series, cartoons, comic books and even theater. Similarly, although three studies explored the effects of storybooks reading on children’s sociocognitive abilities, none of the selected studies portrayed interventions with children’s movies. Finally, it should be noted that most of the selected publications focused on the effects of reading, and only five focused on the effects of movies. In the future, in addition to exploring other types of media, it may be interesting to compare the effects of movies and books in order to assess whether they are similar in intensity and duration of intervention effect.

In summary, the present results indicate that fiction stories, whether presented in movies or literary works, can influence the viewer/reader, depending on different individual characteristics, as well as specific features of the story and the intervention. Further research is needed to better understand the details of this relationship between fiction narratives and social cognition. Even so, the results of this systematic review suggest that the inclusion of fiction movies and books can be a resourceful way of designing interventions to improve social abilities in different groups.
